# Decoding the Histopathology of Pustular Pyoderma Gangrenosum: A Multidisciplinary Approach to Diagnosis

**DOI:** 10.7759/cureus.67059

**Published:** 2024-08-17

**Authors:** Rupali Bavikar, Dipti Singh

**Affiliations:** 1 Pathology, Dr. D. Y. Patil Medical College, Hospital and Research Centre, Dr. D. Y. Patil Vidyapeeth (Deemed to be University), Pune, IND

**Keywords:** pustular dermatosis, pyoderma gangrenosum, parakeratosis, hyperkeratosis, neutrophilic folliculitis, neutrophilic dermatosis

## Abstract

This article discusses the rare neutrophilic dermatosis pustular pyoderma gangrenosum (PG), characterized by necrotizing skin lesions. It highlights the importance of thorough histological examination in diagnosing this variation, which resembles other pustular dermatoses. The case study of a 54-year-old female highlights the unique histological aspects of PG, including epidermal erosion, neutrophilic infiltration, sterile abscesses, and no vasculitis. The article emphasizes the need for differential diagnosis and clinical correlation, emphasizing the importance of collaboration between physicians and pathologists for accurate diagnosis.

## Introduction

Pustular pyoderma gangrenosum (PPG) is a rare, non-infectious, inflammatory neutrophilic dermatosis characterized by painful, aseptic skin ulcers that usually appear after trauma and proceed to a violaceous border. Diagnosis is challenging due to the lack of specific testing and its clinical and histological similarities to other pustular illnesses, making it based on excluding other diseases [1]. Histopathological investigation is crucial for identifying pustular PG and distinguishing it from other illnesses [2]. It involves analyzing tissue samples for histological characteristics like epidermal erosion, neutrophilic infiltration, sterile abscesses, and the absence of vasculitis. These characteristics aid in diagnosing and developing individualized treatment plans, even if they are not pathognomonic, enabling better patient care [3]. Pyoderma gangrenosum (PG) subtypes include classic, peristomal, pustular, bullous, and vegetative, each with unique histologic findings [3]. Histology helps rule out other causes like cancer and infections involving skin and subcutaneous tissue, supporting a clinical diagnosis of PG. A biopsy is typically needed to confirm a PPG diagnosis [4]. This article delves into the complex diagnostic challenges of pustular PG, emphasizing the importance of a thorough histological examination to differentiate it from other neutrophilic dermatoses. It underscores the need for repetitions of collaborative efforts between physicians and pathologists to combine clinical, radiographic, and histological data for accurate diagnosis and effective patient management, thereby enhancing the precision of PG treatment. Such collaborative efforts improve the diagnosis precision and aid in developing successful treatment plans for this uncommon and usually perplexing kind of PG [5].

## Case presentation

**Figure 1:**
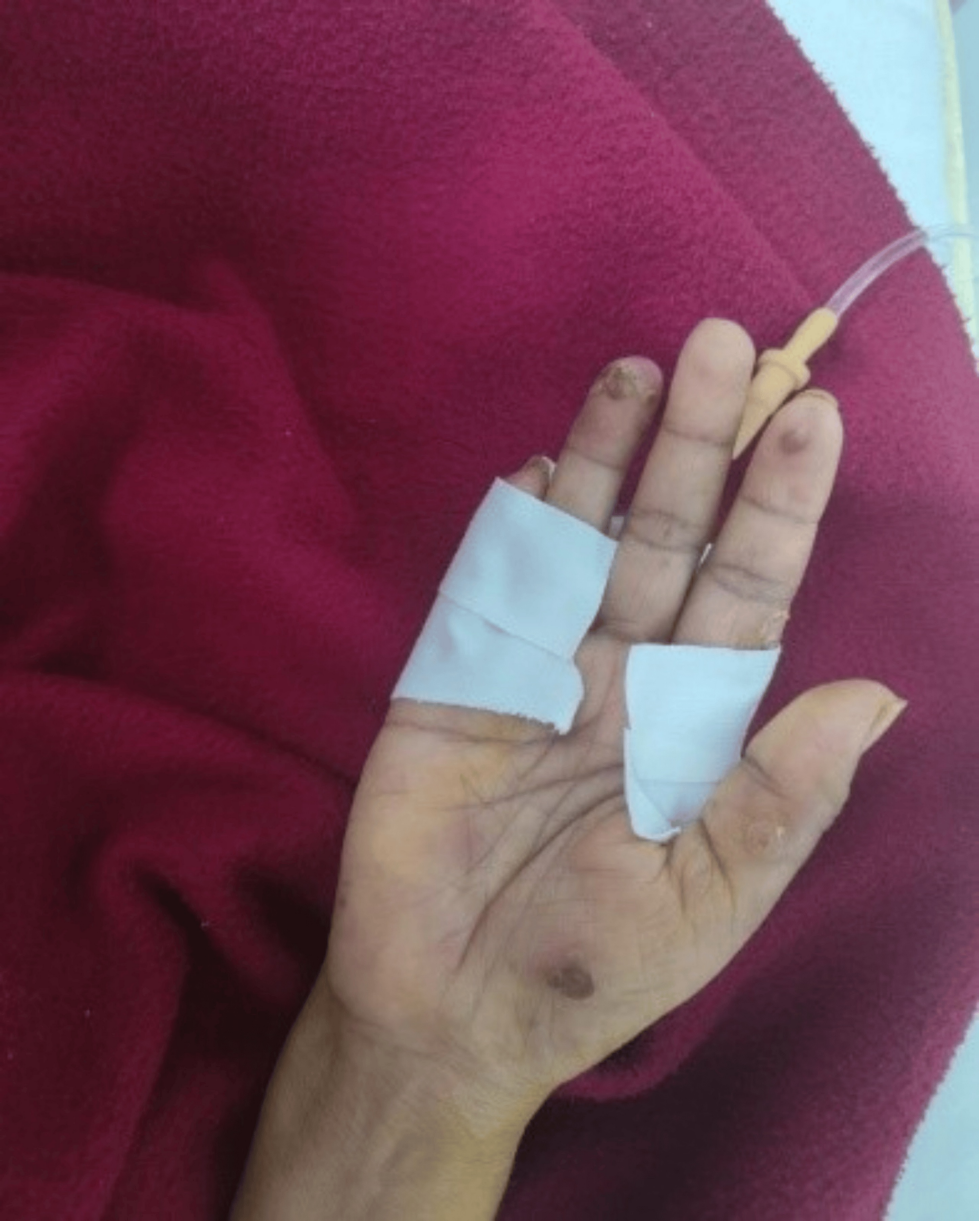
Left upper limb lesion: pustule over the palm Healed lesion at (acral area) fingertips and the thenar part of the hand

**Figure 2:**
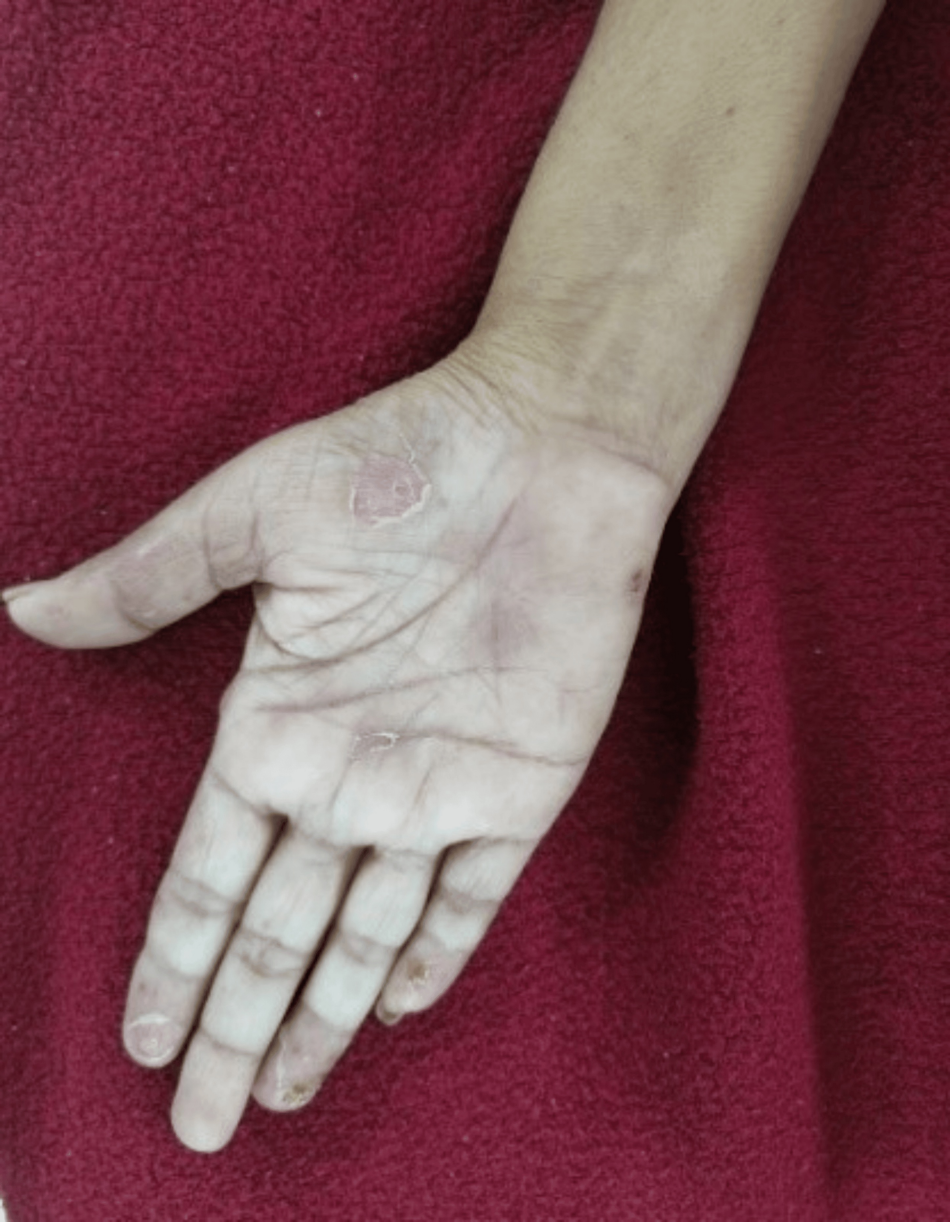
Healed scar on the right palm Healed lesion, leaving a scar at the fingertips and palm (painless)

A 54-year-old female patient reported having dull, excruciating neuropathic pain in her hands and feet that started a few months ago and developed into purulent ulcers, which began as petite, erythematous pustules that were commonly seen on the lower limbs, particularly the legs. They expanded quickly, melding together to create larger ulcers on the right lower leg and both hands (Figures 1-2). Surface ulcers featured violaceous borders and a purulent base. The surrounding skin generally seemed bloated, erythematous, and hot, producing a foul-smelling, scanty, purulent discharge. The patient's additional symptoms were soreness at the ulceration site.

**Figure 3:**
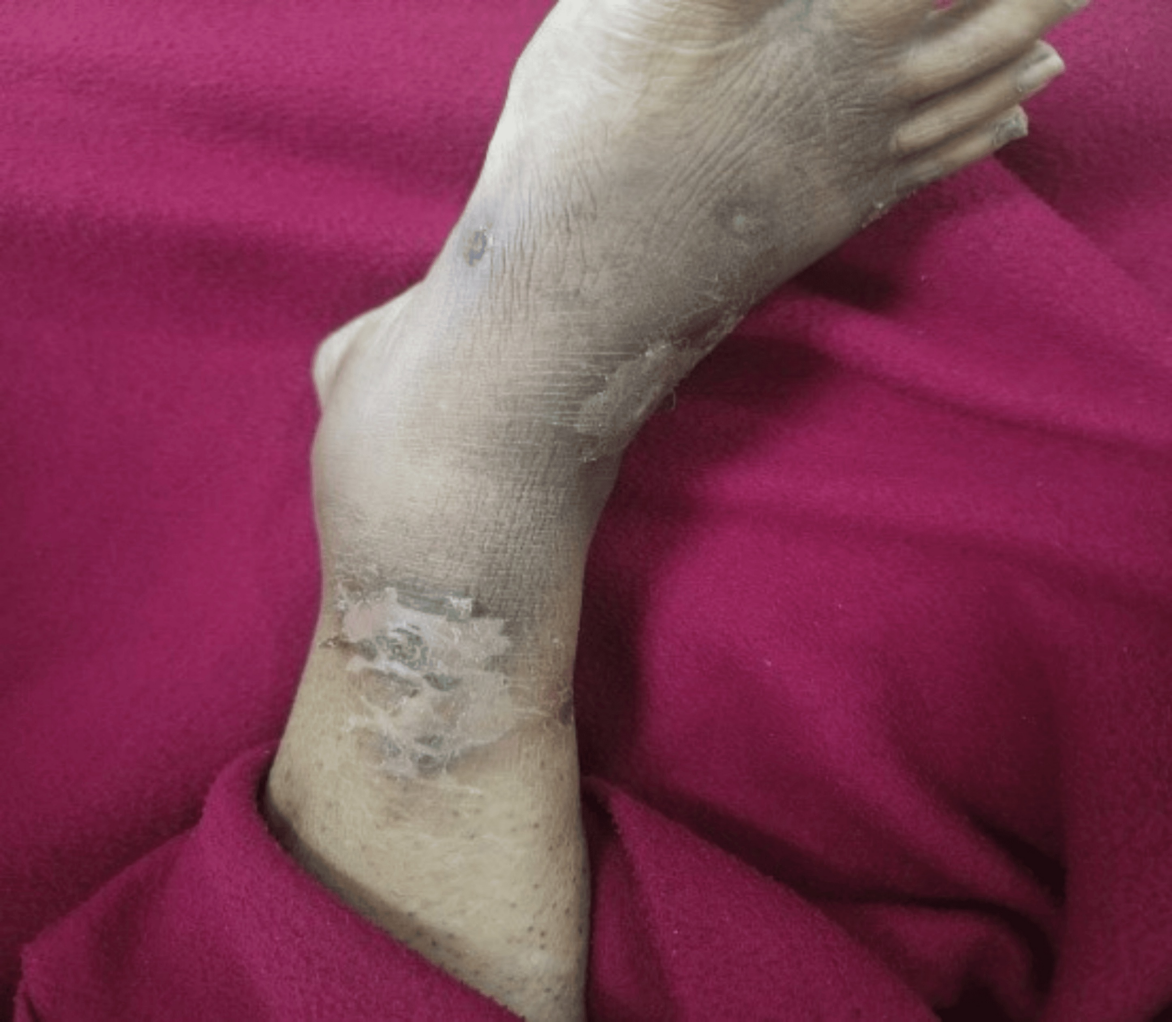
Right lower limb (leg) ulcers Scar lesion at the leg and foot (previously healed)

Local examination revealed recurring blisters on both hands' palmar sides (Figures 1-2), as well as a healed ulcer with scarring on the right leg (a prior lesion) (Figure 3). The surrounding skin was erythematous, violaceous, compromised the integrity of layers, and purulent due to infection. She underwent a colonoscopy for her recurrent abdominal pain, but it did not reveal any significant abnormalities.

Intravenous broad-spectrum antibiotics and hydrocolloid dressings were used as the first treatments but proved ineffective. Her treatment did not involve any immunosuppressants or immunomodulators. The sores kept getting bigger, requiring a biopsy. A 4 mm punch biopsy was taken from the pustules over the left palm (Figure 1). Histopathology was suggestive of a pustular variant of PG. A second colonoscopy was conducted in response to symptoms of intermittent recurrent gastrointestinal pain, and ulcerative colitis was found. Continuous inflammation from the rectum and proximal area caused the mucosa to become erythematous and friable, resulting in an aberrant vascular pattern.

Histopathological findings

**Figure 4:**
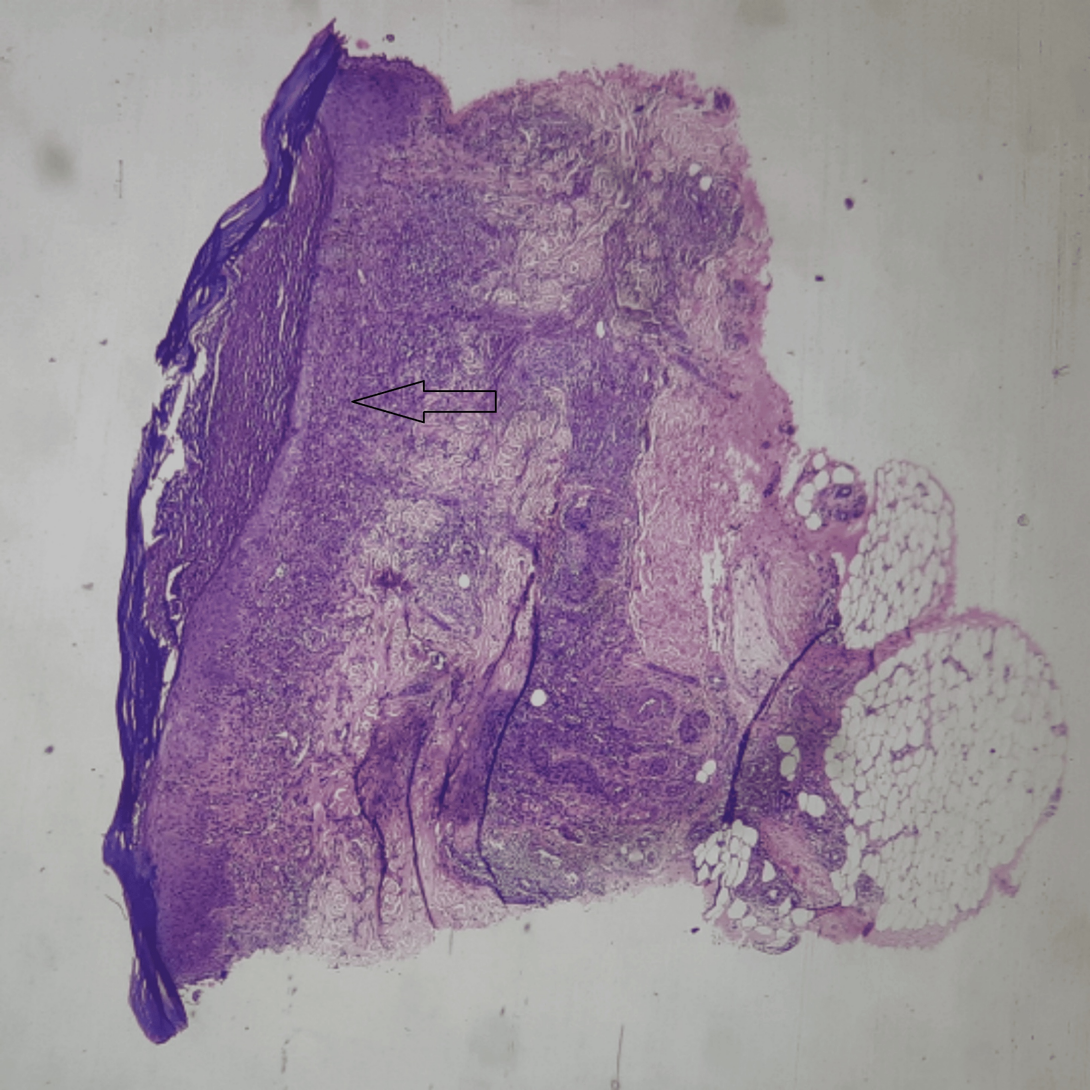
H&E stain on 40x Histopathology of a skin lesion. H&E stain showing full-thickness skin biopsy features of PPG on a 40x scanner (left arrow). A neutrophilic infiltration inside the dermis was found by microscopic inspection, with a preponderance of polymorphonuclear leukocytes. The occurrence of sterile abscesses and epidermis undermining was remarkable. H&E: hematoxylin and eosin, PPG: pustular pyoderma gangrenosum

**Figure 5:**
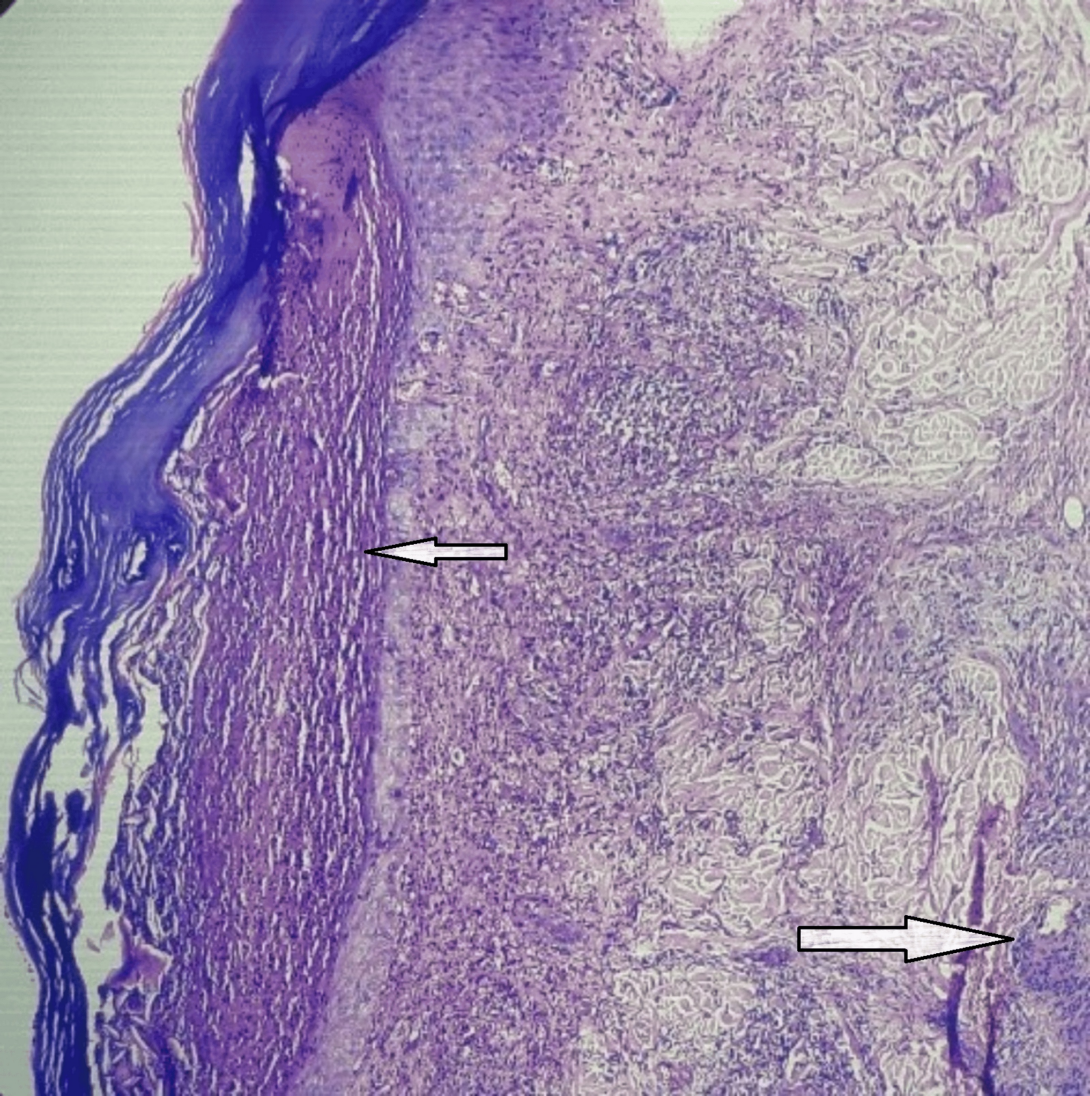
H&E stain on 100x H&E stain on 100x showed the epidermis having hyperkeratosis, parakeratosis, and sub-corneal collection of neutrophils (left arrow). The dermis reveals mixed inflammatory infiltrations around the blood vessels and hair follicles (right arrow). Inflammation is extending up to the deep dermis. Clefts or bullae were not observed. PAS, GMS, ZN, and Gram stains were negative. H&E: hematoxylin and eosin

Microscopic examination showed the epidermis, dermis, and subcutaneous tissue. The epidermis showed hyperkeratosis, parakeratosis, and a sub-corneal collection of neutrophils (Figure 4). Dermis revealed chronic inflammatory infiltration around blood vessels and hair follicles. The blood vessels showed the presence of neutrophils in the vessel wall. Inflammation was extending up to the deep dermis (Figure 5). The biopsy confirmed PPG, with chronic dermal inflammation and neutrophilic abscess, epidermal ulceration, necrosis, mixed inflammatory infiltration, and possible secondary vasculitis, ruling out vascular/neoplastic/SLE and infective etiology. Special stains like PAS, GMS, ZN, and Gram stain were used to rule out infectious processes.

Clinical correlation and treatment

The patient's severe pain, pustules, and ulcers were initially treated with antibiotics and local treatments. The skin biopsy of ulcerative lesions confirmed PG, with epidermal necrosis, neutrophilic infiltration, and mixed inflammatory cells. The repeated colonoscopy examination confirmed ulcerative colitis, characterized by mucosal inflammation and goblet cell depletion, suggesting an underlying inflammatory disorder linked to immune dysregulation. A biopsy and histological investigation identified PPG, leading to a tailored therapy plan. Corticosteroids significantly improved the patient's condition. PPG exacerbations are linked to systemic diseases like rheumatoid arthritis/leukemia/lymphoma or systemic vasculitis, emphasizing the importance of treating underlying issues to effectively regulate PPG. The patient's health improved dramatically with the combination of medicine from a gastroenterologist and a dermatologist.

Histopathology remains the foundation for diagnosing PPG, as it provides definitive information and guides therapy recommendations. The absence of vasculitis distinguishes PPG from similar disorders, emphasizing the importance of histological findings, incorrect diagnosis, and treatment. Pustules or clusters of pustules often form ulcers in the initial stage of PPG. In the early stages, there is a cutaneous abscess and neutrophilic folliculitis/per folliculitis. Later lesions involve a neutrophilic abscess and mixed dermal inflammation. The ulcer grows rapidly, causing tissue necrosis and area expansion. The surrounding skin becomes erythematous, and the edges are violaceous, bluish, and compromised. The ulcers rapidly acquire a purulent layer that smells bad due to a subsequent infection [6,7]. When evaluating ulcerative lesions, direct immunofluorescence (DIF) can be a useful diagnostic technique for ruling out other illnesses such as vasculitis, autoimmune bullous disorders, and other dermatoses that could mimic PG. When paired with clinical and histological evidence, DIF helps restrict the diagnosis to PG by ruling out these differentials.

## Discussion

PPG is a rare neutrophilic dermatosis characterized by epidermal deterioration, significant neutrophilic infiltration in the dermis, and sterile abscesses without vasculitis. Histopathological evaluation is critical for differentiating it from other pustular disorders because it gives important diagnostic indications that guide clinicians to PPG. Common treatments include immunosuppressive medications, wound care, and addressing underlying comorbidities. According to Flora and Frew (2022), neutrophilic infiltration is a crucial characteristic of histology, which is important in verifying suspected instances of PG [5]. Su et al. (1986) supported this by seeing typical pathogenic alterations in PG lesions, such as perivascular lymphocytic infiltration and necrosis [8]. Chia et al. (2008) described a case of pustular PG that was incorrectly identified as necrotizing fasciitis [9], illustrating how challenging it may be to identify PG. Barnes et al. emphasized the significance of retaining a high level of suspicion, especially when treating cases where ulcerative colitis and pustular PG are associated [10]. Maverakis et al. (2020) proposed a diagnostic criterion for PG, focusing on neutrophilic infiltration in skin samples from ulcer margins. A retrospective observational case series study was conducted on 19 patients aged 15-68 years, 13 males and 6 females, from May 2018 to April 2020, to correlate and detect clinically suspected instances [1,11]. Niu et al. (2020) highlighted the link between PG and ulcerative colitis, even in latent cases, in a patient with a large PG requiring multiple medications for healing [12]. Table 1 shows the histological features associated with the PPG-like skin lesions [13].

**Table 1:** Differentials of neutrophilic dermatoses and their histology

Disease	Histological features
Ecthyma	Ulcerated epidermis with fibrinopurulent exudate
	Neutrophilic infiltration in the dermis
	May show epidermal necrosis and crust formation
Sweet's syndrome	Dense neutrophilic infiltration in the dermis (dermal neutrophilic dermatosis)
	Papillary dermal edema and neutrophilic abscesses
	No vasculitis
Erythema multiforme	Interface dermatitis with apoptotic keratinocytes (Civatte bodies)
	Lymphocytic infiltration in the dermis
	May have eosinophils and plasma cells
Bullous pemphigoid	Subepidermal blister formation with eosinophil-rich infiltration
	Linear deposition of IgG and C3 along the basement membrane zone (immunofluorescence)
Dermatitis herpetiformis	Subepidermal vesicles with neutrophils and eosinophils
	Granular IgA deposits at the dermal papillae (immunofluorescence)
	Associated with celiac disease

This study reinforces the importance of histological examination in determining the diagnosis of PG, especially in its pustular form, and the need for a thorough understanding of the lesion's characteristics for accurate diagnosis and treatment planning.

## Conclusions

This case report underlines the necessity of histology in detecting PPG, as well as the need for a multidisciplinary approach. It emphasizes the value of collaboration between physicians and pathologists in integrating clinical and histological data, establishing accurate diagnoses, and customizing treatment modalities. An accurate and timely diagnosis is critical for improved patient care and quality of life.
